# Identification of the RNA polymerase I-RNA interactome

**DOI:** 10.1093/nar/gky779

**Published:** 2018-08-30

**Authors:** David Piñeiro, Mark Stoneley, Manasa Ramakrishna, Jana Alexandrova, Veronica Dezi, Rebekha Juke-Jones, Kathryn S Lilley, Kelvin Cain, Anne E Willis

**Affiliations:** 1 Medical Research Council Toxicology Unit, University of Cambridge, Lancaster Rd, Leicester LE1 9HN, UK; 2 Cambridge Centre for Proteomics, University of Cambridge, Department of Biochemistry, Tennis Court Road, Cambridge CB2 1QR, UK

## Abstract

Ribosome biogenesis is a complex process orchestrated by a host of ribosome assembly factors. Although it is known that many of the proteins involved in this process have RNA binding activity, the full repertoire of proteins that interact with the precursor ribosomal RNA is currently unknown. To gain a greater understanding of the extent to which RNA-protein interactions have the potential to control ribosome biogenesis, we used RNA affinity isolation coupled with proteomics to measure the changes in RNA-protein interactions that occur when rRNA transcription is blocked. Our analysis identified 211 out of 457 nuclear RNA binding proteins with a >3-fold decrease in RNA-protein interaction after inhibition of RNA polymerase I (RNAPI). We have designated these 211 RNA binding proteins as the RNAPI RNA interactome. As expected, the RNAPI RNA interactome is highly enriched for nucleolar proteins and proteins associated with ribosome biogenesis. Selected proteins from the interactome were shown to be nucleolar in location and to have RNA binding activity that was dependent on RNAPI activity. Furthermore, our data show that two proteins, which are required for rRNA maturation, AATF and NGDN, and which form part of the RNA interactome, both lack canonical RNA binding domains and yet are novel pre-rRNA binding proteins.

## INTRODUCTION

The ribosome is a complex macromolecular machine, composed of ribosomal RNA (rRNA) and ribosomal proteins, which translates the genetic code into functional polypeptides. Ribosome biogenesis commences with the synthesis of a large precursor rRNA (pre-rRNA) by RNA polymerase I (RNAPI). Processing of the pre-rRNA through cleavage and trimming events leads to the production of mature 28S, 18S and 5.8S rRNA. Ribosomal proteins are precisely guided on to the folding rRNA to form the pre-40S and pre-60S ribosomal subunits (1). Overall, ribosome biogenesis is a highly coordinated process that requires numerous conserved assembly factors. Thus, in yeast, ∼350 assembly factors have been identified, whereas in mammalian cells, >500 proteins that affect ribosome biogenesis have been described (2,3). Many of these mammalian ribosome biogenesis factors are RNA binding proteins (RBPs). Indeed, recent RNA interactome studies suggest that ∼70% of the 286 proteins that influence rRNA processing may interact with RNA (2). This finding is unsurprising given that the pre-rRNA must be cleaved, digested, modified, and dynamically remodelled to create a functional ribosome. However, it has not been defined which of these potential ribosome biogenesis RBPs contact the rRNA directly and how many have a more indirect role in ribosome biogenesis.

With the advent of RNA interactome capture (RIC) it has emerged that RNA binding proteins are considerably more prevalent than was previously realised (4). One recent estimate suggests that there are nearly 1400 potential RBPs in the human genome (5,6). A substantial number of these RBPs lack classical RNA binding domains and many have no obvious link to RNA biology (5,6). Rather surprisingly, despite the fact that RIC relies on the capture of polyadenylated RNA, a considerable proportion of these RBPs have known roles in ribosome biogenesis or links to nucleolar function. Our data show that RIC also isolates significant quantities of pre-rRNA through RNA-RNA contacts with the mRNA. Therefore, we used this property to characterise those RBPs that directly contact the pre-rRNA or other RNAPI transcripts. We identified 211 potential RBPs that displayed reduced RNA association after RNAPI inhibition, which we designate the RNAPI RNA interactome. Importantly, these RBPs were highly enriched for nucleolar resident proteins and proteins with a known role or proposed role in ribosome biogenesis. Selected proteins from our RNAPI RNA interactome were shown to interact with RNA and these interactions were diminished after RNAPI inhibition. Finally, we extended our study by providing evidence that proteins from the previously identified ANN complex (7), comprised of AATF, neuroguidin (NGDN) and NOL10, and which functions in ribosome biogenesis, are novel pre-rRNA binding proteins.

## MATERIALS AND METHODS

### Cell culture, constructs and stable cell lines

U-2 OS and MCF10A cells were grown at 37°C with 5% CO_2_. U-2 OS were cultured in Dulbecco's modified Eagle's medium (DMEM) supplemented with 10% FBS and MCF10A in DMEM/F12 Ham's Mixture supplemented with 5% horse serum, 10 μg/ml insulin (Sigma-Aldrich), 100 ng/ml cholera toxin (Sigma-Aldrich), 20 ng/ml EGF (Peprotech) and 0.5 mg/ml hydrocortisone (Sigma-Aldrich). MCF10A and U-2 OS cells were treated with 10 nM actinomycin D (Sigma-Aldrich) or 2.5 μM CX5461 (Selleckchem).

The U-2 OS Flp-In T-REx cell line was generated using the manufacturer's recommended protocol (Thermo Fisher Scientific). The coding regions of AATF, NGDN and TAP26 were inserted in frame with the N-terminal 3xFLAG tag in pCDNA5/FRT/TO (Thermo Fisher Scientific). Constructs were then transfected into the U-2 OS Flp-In T-REx cell line and stable clones were selected with 100 μg/ml hygromycin B (Invitrogen) and 2.5 μg/ml blasticidin (Corning).

### Western analysis, immunoprecipitation and RNA immunoprecipitation (RIP)

For western analysis, proteins were separated on 4–12% SDS PAGE gel (Thermo Fisher Scientific), transferred on to PVDF membrane (Bio-Rad) and detected with the indicated antibodies (Supplementary Table S1).

To prepare nuclei, U-2 OS cells were incubated in CLB buffer (10 mM HEPES pH 7.5, 10 mM NaCl, 3 mM MgCl_2_, 0.35 M sucrose, 0.5% NP-40) with protease inhibitors (cOmplete, Roche) for 5 min on ice and then centrifuged at 1300g for 5 min at 4°C. The nuclear pellet was washed twice with CLB and incubated for 30 min in SNEB buffer (20 mM HEPES pH 7.5, 150 mM NaCl, 1% NP40, 0.5% deoxycholate, 0.1% SDS) with protease inhibitors, with or without 0.2 U/ml Benzonase (Novagen), as indicated. The extract was then cleared by centrifugation.

For standard immunoprecipitations, protein G Dynabeads (Thermo Fisher Scientific) were incubated for 30 min with 3 μg of specific antibody (Supplementary Table S1) or rabbit/mouse IgG in TBS. After extensive washing, the antibody-bead conjugates were incubated with protein extract for 2 h at 4°C. Proteins were eluted with either 2xSDS PAGE loading buffer or, for FLAG immunoprecipitations, proteins were eluted with 3 ng/μl FLAG peptide (Sigma-Aldrich).

For RNA immunoprecipitations (RIP), U-2 OS cells were cross-linked with 0.2% formaldehyde for 8 min, incubated in neutralisation buffer (2.5 M glycine and 25 mM Tris pH 7.4) for 5 min and washed twice in PBS. Nuclear extraction and immunoprecipitation were conducted as described above with the following modifications. The beads were washed twice in 20 mM HEPES pH 7.5, 350 mM NaCl, 1% NP40, 0.5% deoxycholate, 0.25% SDS, 5 mM DTT for 3 min and twice with 20 mM Tris pH 7.4, 150 mM NaCl, 0.1% NP-40 for 5 min. Finally, the beads were treated with proteinase K (Thermo Fisher Scientific) and Turbo DNase (Ambion). Purified RNA was used for RT-qPCR analysis.

### RT-qPCR

Reverse transcription was performed with random primers (Invitrogen) and Superscript™ II (Thermo Fisher Scientific) according to the manufacturer's instructions with 5 μl of purified RIP RNA or 300 ng of total RNA. The qPCR was performed using SensiFAST™ Sybr Lo-Rox Mix kit (Bioline) with specific primers to amplify sequences (Supplementary Table S1).

### RNA interactome capture (RIC)

RIC was performed on nuclear extracts of MCF10A and U-2 OS. Briefly, cells were treated with 10 nM actinomycin D, 2.5 μM CX5461 or DMSO. Cells were washed with cold PBS, and cross-linked with UVC (150 mJ/cm^2^) irradiation. Nuclei were isolated as described above and were resuspended in oligo(dT) binding buffer (20 mM Tris pH7.4, 500 mM LiCl, 0.5% LiDS, 1 mM EDTA, 5 mM DTT). Nuclear lysate was incubated with magnetic oligo(dT) beads (NEB) for one hour at room temperature. Subsequently, the beads were washed and the RNA was eluted from the beads (4). The eluted RNA was supplemented with 2 mM MgCl_2_, 125U Benzonase (Sigma-Aldrich) and 300U RNase I (Ambion) to digest the RNA.

### Quantitative mass spectrometry

LC–MS/MS was used to identify and quantify RNA binding proteins (RBPs) as described previously (8). Briefly, UV-crosslinked RBPs were separated by SDS PAGE and serial gel slices digested *in situ* with trypsin (9). Extracted tryptic peptides were analysed using data-independent acquisition (DIA) on a nanoAcquity UPLC system coupled to a Waters Synapt G2-S HDMS mass spectrometer. The PLGS ‘TOP 3’ method was used for absolute quantification of proteins (10).

### Immunolocalization

U-2 OS cells were seeded on to glass coverslips and after the indicated treatments were fixed in 4% paraformaldehyde. Primary antibody incubations were performed at 4°C overnight with the indicated antibodies (Supplementary Table S1). Secondary fluorescent antibodies were incubated for 1 h at room temperature (Supplementary Table S1). Finally, the nuclei were labelled with Hoechst 33342 (Thermo Fisher Scientific). Images were acquired on Zeiss 510 confocal microscope and analysed with ImageJ.

### Bio-informatic analysis

Protein/Gene IDs across different datasets were unified to Uniprot IDs or Gene Symbols using the ‘queryMany’ function in the ‘mygene’ package available through Bioconductor (http://bioconductor.org/packages/release/bioc/html/mygene.html). We assessed our dataset using Gene Ontology, InterPro domain and KEGG pathway annotations all accessed via the ‘mygene’ package (11,12). Analyses were performed using the R statistical framework using the Bioconductor packages goseq, mygene, clusterProfiler and their dependencies (11). ClusterProfiler code was modified to generate easy to interpret visualisation and output tables complete with the genes in each enriched category. The background list used in these analyses was derived from the MCF10A proteome (Dezi, unpublished). The maximum expression of each protein (across replicates) was used as bias during enrichment analyses performed using goseq (13,14). Only those proteins present in the background list are represented in functional enrichment analyses. Hence, there are 457 proteins in our interactome, but only 402 of them are represented in the MCF10A background and have GO terms associated with them. Code used in this study and supplementary data can be accessed via Bitbucket using this link https://bitbucket.org/emm13/2018_pineiro_nucleolarrbp/src.

## RESULTS

### Identification of RNAPI-dependent RNA binding proteins

RNA interactome analysis is a technique that was originally designed to isolate mRNA binding proteins (15,16). First, cells are irradiated with ultraviolet light to covalently couple proteins and any RNA in contact with these proteins *in vivo*. Cells are lysed under denaturing conditions, and polyadenylated mRNA and their associated proteins are then isolated using oligo(dT) affinity chromatography. This isolation process allows for the use of highly stringent wash conditions to remove any non-covalently bound proteins. Consequently, after nuclease digestion to remove the RNA, the sample is highly enriched for proteins that interact with mRNA (17). Recent advances in mass spectrometry have allowed the identification of ‘poly(A) RNA binding proteins’ from several different organisms and cell-types and these studies have vastly expanded the repertoire of potential RBPs (6).

**Figure 1.**
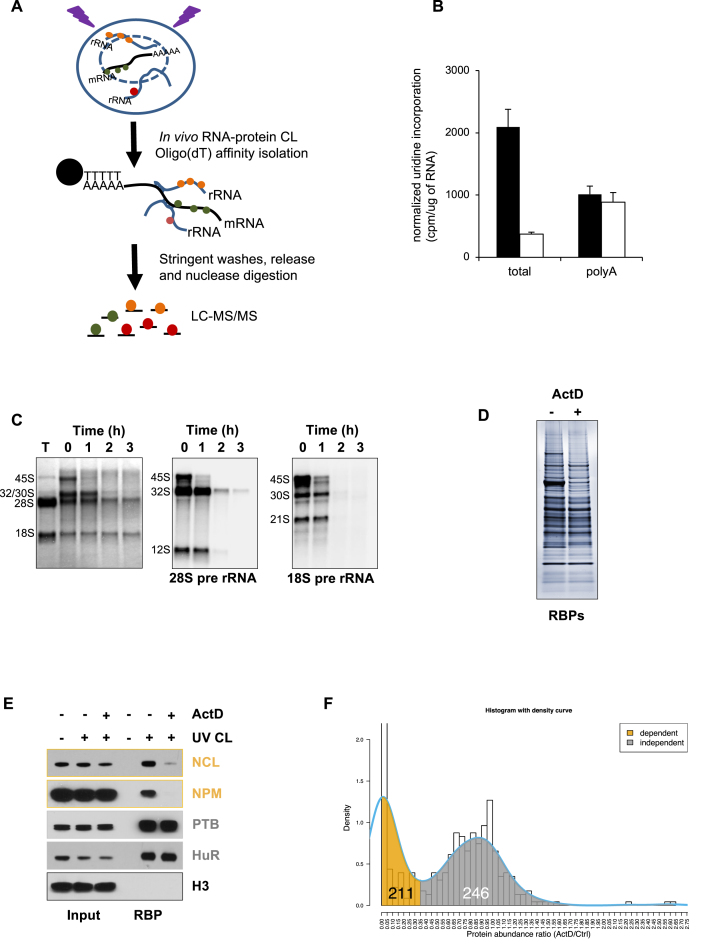
RNA interactome capture to identify RBPs that interact with RNA transcribed by RNAPI. (**A**) Schematic to show the process of RNA interactome capture and how rRNA binding proteins are identified by RIC. (**B**) A low concentration of actinomycin D inhibits total RNA synthesis without affecting mRNA synthesis. Cells were incubated with media containing [^3^H]-uridine and total RNA or polyadenylated mRNA was isolated. After normalising to the amount of RNA, the radioactivity incorporated into the two fractions was taken as a measure of RNA synthesis. Black bars are control samples and white bars are ActD-treated samples. (**C**) RNA isolated by RIC contains substantial amount of rRNA species. RIC was performed and the resulting RNA was analysed by northern analysis (35). Lane 1, 'T" denotes total cellular RNA (nucleus and cytoplasm), lanes 2–5 are RIC RNA prepared from the nuclear RNA fraction. Methylene blue staining shows significant quantities of mature rRNA and pre-rRNA in the RIC RNA. The 28S and 18S pre-rRNA intermediates were detected using radiolabelled oligonucleotides to ITS1 and ITS2 (35). Treatment of the cells with 10 nM actinomycin D results in a time-dependent loss of pre-rRNA from the RIC RNA. (**D**) RNA interactome capture was performed on control and actinomycin D-treated MCF10A cells. The resulting proteins were separated by SDS PAGE and visualised using silver staining. (**E**) RIC was performed as in (D) and the RNA binding proteins nucleolin (NCL), nucleophosmin (NPM), polypyrimidine tract binding protein (PTB) and ELAV-like protein 1 (HuR) were detected using western analysis. The RBPs can be detected only after UV cross-linking. Actinomycin D treatment significantly reduces the recovery of the pre-rRNA binding proteins NCL and NPM, but has no effect on the mRNA binding proteins PTB and HuR. Histone H3 (H3) was used as a non-RNA binding control. (**F**) Mass spectrometry analysis identified 457 RBP candidates. The figure shows the distribution of the ratio of protein abundance between ActD treated and control cells. The density curve (blue) outlines the trend of the distribution showing two peaks at 0 (complete loss after ActD treatment) and at 0.9 (very little loss after ActD treatment). The superimposed shading under the density curve shows the cut-off used to determine RNAPI-dependent (gold, ratio ≤ 0.35) and RNAPI-independent (gray, ratio > 0.35) proteins.

In preliminary experiments using RIC, we noted that the RNA that was isolated using this technique contained a significant quantity of both pre-ribosomal RNA and mature rRNA (Supplementary Figure S1 and Figure 1C). Since the buffer conditions in the RIC protocol (0.5% LiDS, 0.5% LiCl) favour nucleic acid hybridization, we reasoned that rRNA was purified as a consequence of interactions between the mRNA and the abundant rRNA (Figure 1A). Disruption of these RNA-RNA interactions using heat coupled with two rounds of oligo(dT) purification, as used in the original RIC protocol from the Dreyfuss lab (18), resulted in almost complete loss of rRNA species from the RIC RNA, but the majority of the mRNA was retained (Supplementary Figure S1B). Moreover, we show that prior depletion of poly(A) RNAs from the lysate results in complete loss of rRNA species in the RIC RNA (Supplementary Figure S1C). Thus, these data suggest that the more recently developed RIC protocol, which uses only one round of oligo(dT) purification and no denaturation of the RNA (17), has the potential to identify not only mRNA binding proteins, but also rRNA binding proteins (Figure 1A).

Since an RNA interactome contains pre-ribosomal RNA (Supplementary Figure S1; Figure 1C), we reasoned that we could identify RBPs involved in ribosome biogenesis by analyzing changes in the RNA interactome that occur after *in vivo* pre-rRNA depletion through inhibition of RNAPI. We focussed on the nuclear RNA interactome to minimize the potential for RNA-protein interactions in the mature ribosome masking any changes that are due to the loss of pre-rRNA and we used a low concentration of actinomycin D (ActD) to preferentially inhibit rRNA transcription (19). Thus MCF10A cells were exposed to ActD at a concentration that was sufficient to inhibit the activity of RNAPI, but with minimal effect on RNA polymerase II (RNAPII) transcription. Three hours after exposure to 10 nM ActD, we confirmed that there was a substantial decrease in total RNA transcription (Figure 1B). Furthermore, northern analysis of RIC RNA revealed that there was a large decrease in the abundance of the 45S, 32S, 30S and 12S pre-rRNA in the captured RNA, indicating that rRNA transcription was inhibited under these conditions and that most of the existing intermediates are processed during this time (Figure 1C). However, ActD had little or no effect on the synthesis of polyadenylated RNA and therefore at this concentration the drug has little effect on RNAPII transcription (Figure 1B). Subsequently, RBPs were isolated by RIC from control and ActD treated cells. The spectrum of recovered RBPs was altered considerably after RNAPI inhibition, suggesting that many RBPs recovered by RIC interact with RNAPI RNA (Figure 1D). Examination of the recovered RBPs by western analysis revealed that, as we predicted, the association of the known pre-rRNA binding proteins, nucleolin (NCL) and nucleophosmin (NPM) with RNA, was considerably reduced after RNAPI inhibition. However, the recovery of the mRNA binding proteins PTB and HuR was unchanged under these conditions (Figure 1E). These observations strongly suggested that our method selectively depletes RNAPI RNA and its associated RBPs and therefore this approach could be used to characterise the RNAPI RNA interactome.

RBPs were isolated from control and ActD treated cells followed by quantitative label-free mass spectrometry to reveal RNA binding interactions that are dependent on RNAPI activity. We identified 457 potential RNA binding proteins in the nuclear proteome of MCF10A cells (Supplementary Table S2). Of these proteins, 211 showed reduced binding (3-fold or more; ActD/Ctrl ratio ≤ 0.35) following treatment with ActD and we classified these as RNAPI-dependent RBPs (RNAPI-dep; Figure 1F). Those proteins that do not show a significant change after RNAPI inhibition (246/457) were labelled as RNAPI-independent RBPs (RNAPI-indep; Figure 1F, Supplementary Table S2).

**Figure 2.**
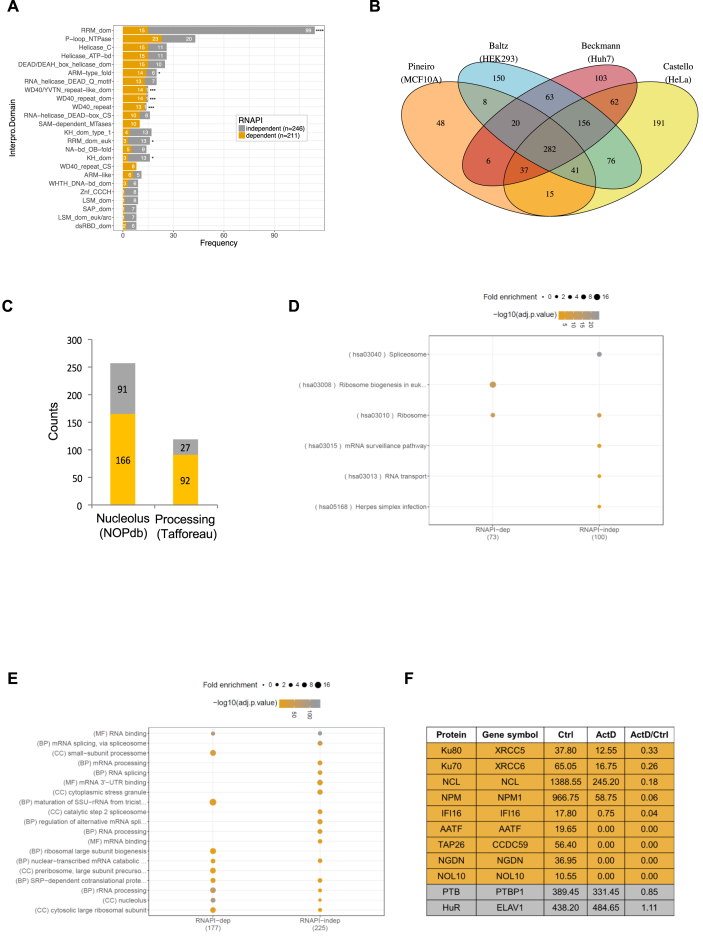
Bio-informatic analysis of the MCF10A RNA interactome. (**A**) All proteins in the RNAPI-dependent and independent lists were mapped to Interpro domains (https://www.ebi.ac.uk/interpro/). A barplot was generated showing the distribution of the most frequently occurring (in 5 or more proteins) InterPro domains for both lists. (**B**) A Venn diagram showing the overlap of this study, Pineiro (orange), with three other RNA interactome capture studies – Baltz (blue), Beckmann (red) and Castello (yellow). (**C**) RNA interactome data were compared to nucleolar proteins from Andersen *et al*. ((21); ‘NOPdb’) and rRNA-processing proteins from Tafforeau *et al* ((2); ‘Tafforeau’). Of the proteins in our study that overlapped with either NOPdb or Tafforeau, we further classified them into RNAPI-dependent (gray) or RNAPI-independent (gold/yellow). These counts are displayed as stacked barplots for the two studies. (**D**) RNAPI-dependent and RNAPI-independent RBPs were annotated using Kyoto Encyclopaedia of Genes and Genomes (KEGG) pathways (https://www.genome.jp/kegg/). Following annotation, an enrichment analysis was performed against a background of MCF10A proteins. The size of the dot represents the fold enrichment over the background. The colour of the dot represents the significance of the enrichment (negative log2 of the adjusted *P*-value) with grey being most significant and orange being the least. All terms displayed are significant and above the *P*-value cut-off of 0.05. See Supplementary Table S7 for a full list. (**E**) All proteins in the RNAPI-dependent and independent lists were mapped to Gene Ontology (GO) terms (http://www.geneontology.org/). An enrichment for GO terms was performed against a background of the MCF10A proteome. The plot shows a visualisation of GO enrichment analysis for the proteins in RNAPI-dependent and RNAPI-independent lists. The numbers in brackets represent the number of proteins in the input list that could be mapped to GO categories – 177/211 for RNAPI dependent and 225/246 for RNAPI-independent proteins. The Y-axis shows the top GO categories represented in the dataset. The size and colour of dot represent fold enrichment and significance as in (D). Note that all terms displayed are significant and above the *P*-value cut-off of 0.05. (**F**) A table showing a subset of proteins identified in the study that were chosen for further experimental analysis ordered by RNAPI-dependent proteins (orange) in decreasing order of ActD/Ctrl ratio. Control RNAPI-independent, (mRNA-binding proteins) are displayed in grey. Candidate RNAPI-dependent RBPs have a ratio of 0.35 or lower (>3-fold decrease in binding to RNA when cells are treated with actinomycin D) in our mass spec analysis, whereas the control RBPs have a ratio close to 1.

Analyses of these data showed that 384 of 402 proteins with Gene Ontology (GO) terms (95%) are functionally annotated as RNA binding (Supplementary Table S3: ‘GO:0003723’). As expected, many of the RBPs identified contain canonical RNA binding domains such as RRM and KH domains (Figure 2A) and interestingly, a significantly higher proportion of RNAPI-independent RBPs containing the RRM domain relative to their RNAPI-dependent counterparts (Figure 2A; *P* value = 1.562e–15). Furthermore, a comparison of the MCF10A RNA interactome capture data with those from three other cell lines (17,20) showed that 90% (409/457) of these candidate RBPs have been identified previously using RIC (Figure 2B). A substantial number of the remaining 10% (48/457) also have RNA binding functions (red circles, Supplementary Figure S2A). In conjunction, these analyses confirm that our method has delivered a high confidence set of RNA binding proteins.

### Functional analysis supports RNAPI-dependent RBPs as nucleolar resident proteins.

We predicted that the majority of nuclear RBPs that have decreased RNA binding upon RNAPI inhibition are located in the nucleolus. Using GO annotations, 193 of the 457 nuclear RBPs are known to be resident in the nucleolus (‘GO:0005730’) of which 75% (144) are RNAPI-dependent (Supplementary Table S2). In addition, 56% (257/457) of the nuclear RBPs identified in this study were present in the nucleolar proteome characterised by Andersen *et al*. (21) with 79% (166/211) of the RNAPI-dependent and 37% (91/246) of the RNAPI-independent RBPs described as nucleolar (Figure 2C, Supplementary Tables S4 and S5). To gain further support for the nucleolar localisation of the RNAPI-dependent RBPs, we searched for the presence of nucleolar localisation signals (NoLS; (22)) and nucleolar detention signals (NoDS; (23)). Our data showed no difference between NoDs in the RNAPI-dependent versus independent RBPs. However, there was a significant difference in the distribution of NoLS, with more NoLS decorating RNAPI-dependent proteins than RNAPI-independent proteins (Supplementary Figure S2B). Taken together these observations support the nucleolar localisation of the RNAPI-dependent RBPs.

To gain insight into the function of the nuclear RBPs, we performed enrichment analyses using Kyoto Encyclopedia of Gene and Genomes (KEGG) pathways and GO terms (Figure 2D and E; Supplementary Table S3 and S6). KEGG pathway analysis revealed that the RNAPI-dependent RBPs are enriched for proteins involved in ribosome biogenesis, whereas the RNAPI-independent RBPs play a role in splicing, mRNA surveillance and RNA transport (Figure 2D). When GO enrichment was applied to the RNAPI-dependent proteins, the terms identified are rRNA processing (GO:0006364), maturation of SSU-rRNA from tricistronic rRNA transcript (GO:0000462) and ribosomal large subunit biogenesis (GO:0042273) with gene products associated with the nucleolus, the small subunit processome and the large subunit pre-ribosome (Figure 2E; Supplementary Table S6). In addition, 44% (92/211) of the RNAPI-dependent RBPs were also identified as playing a role in ribosomal RNA processing in a large scale siRNA screen (2), whereas, only 10% (27/246) of the RNAPI-independent RBPs scored positive in this screen (Figure 2C). Thus, the RNAPI-dependent RBPs are highly enriched for ribosome biogenesis functions. In contrast, the biological processes associated with the RNAPI-independent RBPs are mRNA splicing (GO:000398), mRNA processing (GO:006397) and the regulation of alternative splicing (GO:000381) (Figure 2E).

We chose to investigate further RNAPI-dependent RBPs that have no canonical RNA binding domains and have a poorly characterised role in ribosome biogenesis (Figure 2F). Thus, AATF, NOL10 and NGDN are part of the recently described ANN complex that may play a role in pre-rRNA cleavage (7). TAP26 and IFI16 were previously identified as potential ribosome biogenesis factors (2). Finally, the Ku proteins (KU70/80) are known to be nucleolar and interact with the telomerase RNA (hTR) (24,25), but they have never been defined as ribosome biogenesis factors. All of these candidate RNAPI-dependent RBPs were identified in previous RNA interactome analyses, supporting our observations that they contact RNA. Nevertheless, there is no direct evidence that any of these proteins interact with pre-rRNA.

### RNAPI-dependent RBPs accumulate in the nucleolus

In the first instance, we analysed the subcellular distribution of our chosen proteins to confirm that they accumulate in the nucleolus. For immunolocalisation of AATF, KU70, NOL10 and IFI16, we used antibodies to the endogenous proteins. Commercial antibodies of sufficient quality were not available for immunolocalisation of NGDN or TAP26 and therefore we generated stable inducible cell lines expressing epitope tagged proteins using the Flp-In T-REx system (Supplementary Figure S3). All of our subsequent experiments were performed in U-2 OS cells since we encountered problems establishing a stable Flp-In MCF10A cell line. We felt justified in doing so because ribosome biogenesis is an extremely well conserved process and U-2 OS are a common model cell line to study nucleolar functions. In addition, large-scale protein localisation data are available for these cells as part of the Human Atlas protein project (26). We also investigated the role of RNAPI transcription on the nucleolar retention of the RNAPI-dependent RBPs using the RNAPI inhibitors ActD and CX5461. CX5461 is a selective inhibitor of RNAPI that prevents the polymerase from loading on to the rDNA promoter (27,28). We used ActD and CX5461 at concentrations that substantially inhibited rRNA synthesis, but had no effect on mRNA synthesis. U-2 OS cells were treated with either CX5461 or ActD and rRNA transcription was measured using RT-qPCR analysis of precursor rRNA that contain the 5′ETS. Treatment of cells with CX5461 or 10 nM ActD substantially inhibited rRNA transcription, whereas the abundance of the short-lived c-*myc* mRNA was unaltered by these treatments (Supplementary Figure S4).

**Figure 3.**
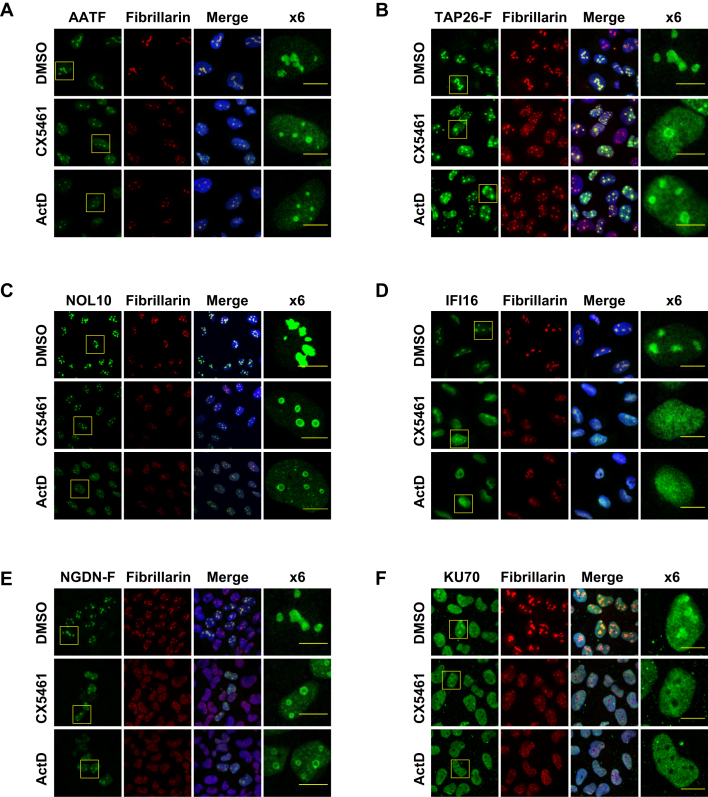
Subcellular distribution of AATF, TAP26, NOL10, IFI16, NGDN and KU70 in control and ActD or CX5461-treated cells. Immuno-localisation of AATF (**A**), FLAG-tagged TAP26 (**B**), NOL10 (**C**), IFI16 (**D**), FLAG-tagged NGDN (**E**) and KU70 (**F**) in U-2 OS cells. Fibrillarin and Hoechst staining were used as nucleolar and nuclear markers respectively. The subcellular distribution of each protein was monitored in control cells and in cells treated with actinomycin D (ActD) or CX5461. Each of the candidate RNAPI-dependent RBPs is present in the nucleolus. The size bar represents 10 microns.

In unperturbed cells, the majority of AATF, TAP26, NOL10, IFI16 and NGDN was found in the nucleolus (Figure 3A–E). In contrast, as reported previously (24), KU70 was found predominantly in the nucleoplasm with some accumulation in the nucleolus (Figure 3F). After inhibition of RNAPI transcription with ActD or CX5461, KU70 staining was no longer found in the nucleolus, suggesting that RNAPI activity facilitates the retention of KU70 in the nucleolus (Figure 3F). A significant quantity of TAP26, IFI16 and NGDN was also released from the nucleolus after RNAPI inhibition (Figure 3B, D, E). However, ActD and CX5461 had little or no effect on the nucleolar localisation of NOL10 or AATF (Figure 3A and C). We speculate that in the case of proteins that remain associated with the nucleolus following RNAPI inhibition such as AATF and NGDN, both RNA-protein and protein-protein interactions are required for tethering in this organelle. However, proteins that are located in the nucleolus solely due to rRNA binding (e.g. IFI16 and KU70) are released following exposure to cell stresses that inhibit RNAPI allowing their relocation into the cytoplasm/nucleoplasm, where they may have alternative functions. Thus, all of our candidate RNAPI-dependent RBPs have a nucleolar presence, but the extent to which nucleolar accumulation depends on RNAPI transcription varies between proteins.

### The interaction of AATF, NOL10, NGDN, IFI16, TAP26, KU70 and KU80 with RNA is disrupted following inhibition of RNAPI

**Figure 4.**
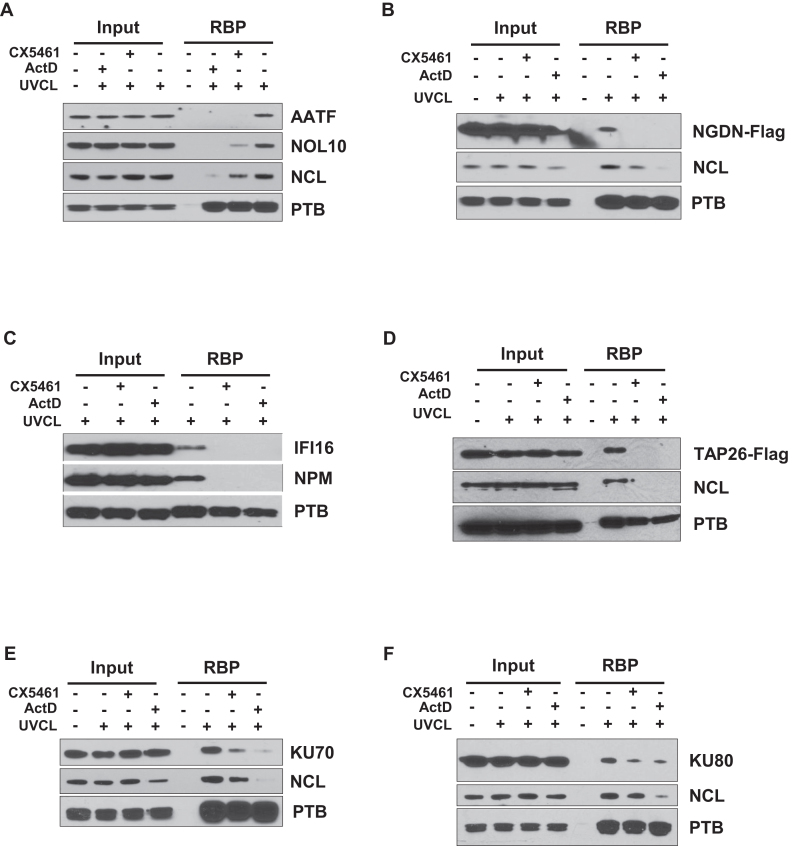
Candidate RNAPI-dependent RBPs show reduced binding to RNA following inhibition of RNAPI. RNA interactome capture was performed in cells treated with the RNAPI inhibitors ActD or CX5461 and in untreated cells. Samples were separated by SDS PAGE and subjected to western analysis using antibodies against AATF and NOL10 (**A**), NGDN (**B**), IFI16 (**C**), TAP26 (**D**), KU70 (**E**) and KU80 (**F**). The data confirm that the recovery of the candidate RNAPI-dependent RBPs decreases after RNAPI inhibition. Nucleolin (NCL) and nucleophosmin (NPM) were used as pre-rRNA binding protein controls and PTB was used as an mRNA binding protein control.

To validate the RNAPI RNA interactome, we inhibited RNAPI activity using ActD or CX5461 and performed RNA interactome capture followed by western analysis for our proteins of interest. Nucleolin (NCL) or nucleophosmin (NPM), which are RBPs with well-described roles in ribosome biogenesis, were used as positive controls, and the mRNA binding protein, PTB, was used as a negative control. After inhibition of RNAPI, there was a considerable reduction in the recovery of AATF, NOL10, NGDN, TAP26 and IFI16 in the RIC samples and a more modest reduction in the recovery of the Ku proteins (Figure 4). The magnitude of the changes is in complete agreement with our mass spectrometry experiments (Figure 2F). The pre-rRNA binding proteins NCL and NPM also show decreased recovery in these experiments, whereas the recovery of mRNA binding protein PTB is unchanged. Taken together these data suggest that our candidate proteins interact with RNAPI RNA. For AATF, NOL10, NGDN, IFI16 and TAP26, inhibition of rRNA synthesis has a substantial impact on the recovery of these proteins by RIC, suggesting that the major RNA species that these proteins interact with in this assay is derived from RNAPI (Figure 3A–D). However, the partial inhibition of Ku protein RNA binding after RNAPI inhibition (Figure 3E and F) most likely reflects the interaction of these proteins with other classes of RNA, in addition to RNAPI transcripts (29,30).

### AATF and NGDN are novel pre-rRNA binding proteins

To provide direct evidence for the interaction of proteins identified in our RNAPI interactome with ribosomal RNA we chose to study AATF and NGDN in further detail. It has been suggested that these proteins in combination with NOL10 form a complex, the ANN complex, that plays a role in 18S rRNA maturation by facilitating endonucleolytic cleavage within the 5′ETS and ITS1 (7). Our RNA interactome data confirms that all three proteins contact RNA derived from RNAPI, despite the lack of a recognizable RNA binding domain in any of the three proteins. Clearly, the major RNAPI transcript in the cell is pre-rRNA and therefore, we tested the hypothesis that the members of the ANN complex are novel pre-rRNA binding proteins.

**Figure 5.**
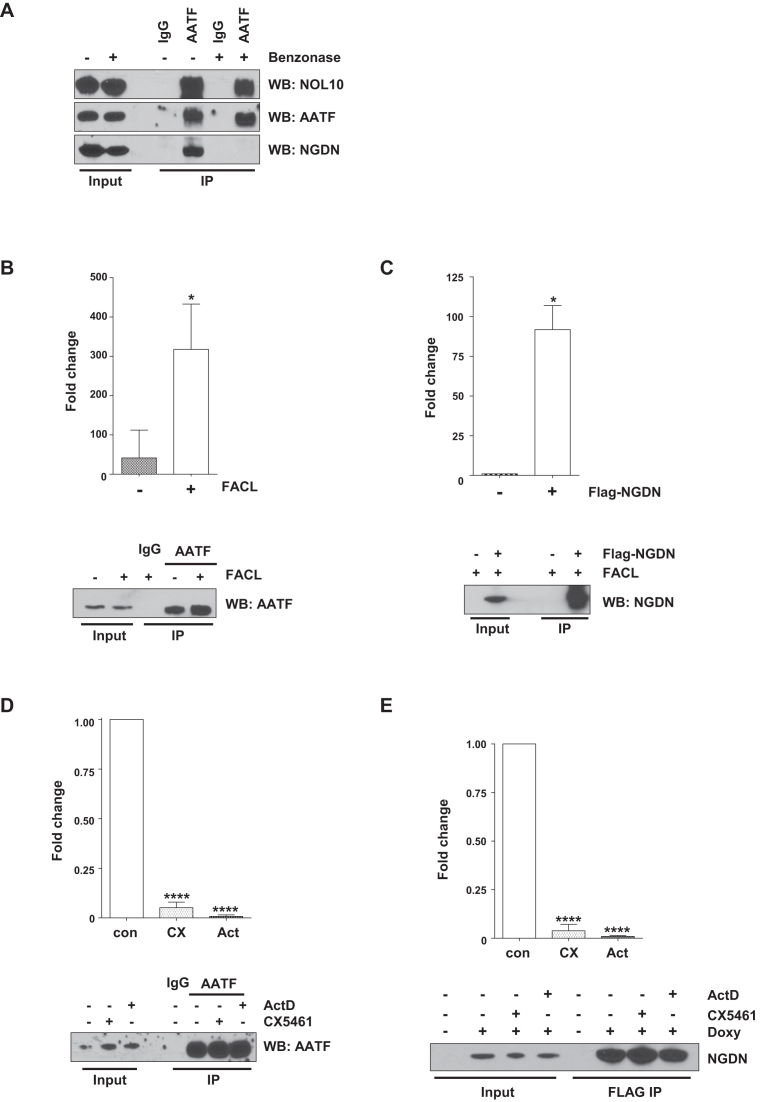
ANN complex RBPs AATF and NGDN bind to pre-rRNA. (**A**) AATF was isolated from U-2 OS nuclear extracts by immunoprecipitation using a specific antibody. IgG was used as a negative control. Immunoprecipitations were performed from extracts prepared either with or without Benzonase treatment. The immunoprecipitated proteins were separated by SDS PAGE and AATF, NGDN and NOL10 were detected by western analysis. The data show that AATF forms a complex with NOL10 and NGDN, but that the presence of NGDN in the complex depends on nucleic acid. (**B**) AATF was immunoprecipitated from nuclear extracts prepared from untreated and formaldehyde cross-linked U-2 OS cells. In addition, control immunoprecipitations using IgG were performed. Samples were subjected to western analysis to confirm the specific isolation of AATF. In parallel, RNA was isolated from immunoprecipitates and RT-qPCR was carried out using primers to detect rRNA precursors. The y-axis on the graph represents the average fold enrichment of rRNA precursors containing the 5′ETS in the different conditions relative to the IgG control. Values are the mean ± SD of three independent experiments. * (*P* < 0.05) denotes statistically significant differences in RNA levels. (**C**) Cross-linking RNA immunoprecipitation of NGDN using the NGDN FLAG-tagged cell line. Immunoprecipitation was performed in control (–) and doxycycline-treated (+) cells using the FLAG antibody. Western analysis and qRT-PCR analysis was carried out as described in (B). Y-axis values are the mean ± SD of three independent experiments. * (*P* < 0.05) denotes statistically significant differences in RNA levels. (**D**) U-2 OS cells were treated with CX5461 (CX) or ActD (Act), followed by *in vivo* formaldehyde cross-linking. AATF was immunoprecipitated and protein samples were subjected to western analysis. The RNA isolated from the AATF immunoprecipitations was subjected to RT-qPCR with specific primers as described above. The data represent the mean ± SD of three independent experiments, **** (*P* < 0.0005). (**E**) Cross-linking RIP was performed on cells treated as described above, using FLAG antibodies to isolate FLAG-tagged NGDN. Protein samples and RNA samples were processed as described in (D). The data represent the mean ± SD of three independent experiments, **** (*P* < 0.0005).

Initially, we attempted to confirm the previously published interaction between these three proteins (7). When endogenous AATF was immuno-affinity isolated from U-2 OS cells, we found that significant quantities of NOL10 and NGDN co-purified with this protein, suggesting that the three proteins are present in a complex (Figure 5A). Interestingly, only NOL10 co-purified with AATF when AATF was immuno-affinity purified from nuclease-treated extracts (Figure 5A). The release of NGDN from the complex following nuclease treatment implies that NOL10 and AATF interact through protein-protein interactions, whereas NGDN is co-purified with AATF and NOL10 through RNA–protein interactions.

To probe the interaction of AATF and NGDN with pre-rRNA, we took a cross-linking immunoprecipitation approach. Thus, RNA-protein complexes were cross-linked *in vivo* using a short exposure to a relatively low concentration of formaldehyde. Subsequently, immuno-affinity isolation was performed with stringent lysis and wash conditions to selectively purify cross-linked RNA-protein complexes. After immunoprecipitation, the association of AATF and NGDN with pre-rRNA was investigated by RT-qPCR amplification of the 5′ETS pre-rRNA sequence. In the absence of cross-linking, pre-rRNA is enriched in the AATF immunoprecipitation by ∼40-fold compared to the IgG control. However, after cross-linking there is a 315-fold enrichment of pre-rRNA in the AATF immunoprecipitation (Figure 5B). These data indicate that we have selectively enriched cross-linked RNA-protein complexes under these conditions and that AATF interacts with the pre-rRNA. In addition, under the same conditions we observed a 92-fold enrichment of pre-rRNA after cross-linking immunoprecipitation of NGDN (Figure 5C). Therefore, NGDN also interacts with the pre-rRNA. We further confirmed the specificity of the interaction of AATF and NGDN with pre-rRNA by inhibiting the synthesis of pre-rRNA with either ActD or CX5461 and performing cross-linking immunoprecipitation RT-qPCR. The abundance of pre-rRNA in both AATF and NGDN cross-linked immunoprecipitations decreased dramatically after inhibition of RNAPI, confirming that AATF and NGDN specifically interact with the pre-rRNA (Figure 5D and E). When combined with our RNA interactome study, these data confirm that AATF and NGDN are novel pre-rRNA binding proteins.

## DISCUSSION

RNA interactome capture was originally developed to identify novel mRNA binding proteins (18), but importantly our data show that it can also be used to identify RBPs that associate with other RNA species. Thus, we demonstrate that the same conditions that favour interaction between oligo(dT) and polyadenylated RNA also drive the interaction of an abundant RNA, such as the pre-rRNA, with the captured polyadenylated RNA (Figure 1A, Supplementary Figure S1). Therefore, we exploited this phenomenon to define the RBPs that associate with RNAPI RNA in the nucleus. We identified 211 RBPs with a >3-fold difference in RNA binding following the selective inhibition of RNAPI activity (Figures 1 and 2) and we have designated these RBPs as the RNAPI RNA interactome. Our data have important implications for the interpretation of the previously published RNA interactome capture experiments. We suggest that these data cannot be regarded as mRNA interactomes or even poly(A) RNA interactomes, but that they should be considered to be RNA interactomes. This conclusion is supported by analysis of the composite list of human RNA interactomes (6), which reveals that 87% (183/211) of our RNAPI-dependent RBPs are present in this list (Supplementary Table S6). Furthermore, a comparison of the composite list with the nucleolar protein database (NOPdbv2 (21)) reveals that 32% (442/1393) of these proteins are nucleolar (Supplementary Table S7).

An alternative strategy to identify RBPs associated with a specific transcript is to use oligonucleotides-linked to beads that target a defined RNA. For example, the recently developed method by Rogell *et al*., whereby proteins bound to the target RNA are captured by hybridization with antisense locked nucleic acid (LNA)/DNA oligonucleotides covalently coupled to a magnetic resin (31). However, although this technique was successfully employed to capture the RBPs bound to a reporter mRNA containing the Sex-lethal (Sxl) binding motifs, when LNAs were directed against 18S or 28S rRNAs very few ribosomal proteins were obtained, with <30 proteins identified. As expected, there is limited overlap between our data and the data of Rogell *et al*., however, many of the ribosomal RNA binding proteins identified by Rogell *et al*. were classified by our work as RNAPI independent (e.g. HNRNPs, YBX3, ELAV1), suggesting that some of these interactions may not be rRNA specific. The authors suggested that the dearth of ribosomal proteins identified was likely to be due to inefficient crosslinking of proteins bound to double-stranded RNA, which involves much of the ribosomal RNA. Importantly, our data show that the many interaction sites provided by mRNA-rRNA contacts in the RIC technique are sufficient to overcome such issues and the method that we use provides an accurate data set of the RBPs that interact with rRNA.

RNAPI is known to generate two species of RNA, the 45S pre-rRNA and non-coding RNA derived from the intergenic spacer region of rDNA (IGS rRNA) (32). IGS rRNA are approximately 100-fold less abundant than pre-rRNA (32), suggesting that the majority of the RNA-protein interactions we observe in the RNAPI RNA interactome involve pre-rRNA. Consistent with this interpretation, bioinformatic analysis reveals that terms associated with ribosome biogenesis are significantly enriched in our RNAPI-dependent RBPs (Figure 2) and ∼73% of these proteins have previously been shown to influence ribosome biogenesis (2,3). Thus, characterisation of the RNAPI RNA interactome has provided a comprehensive record of the RBPs that influence ribosome biogenesis, particularly those involved in the processing of pre-rRNA. Furthermore, we have now confirmed that AATF and NGDN, which have been shown to play a role in facilitating cleavage of the pre-rRNA within the 5′ETS and ITS1 (7), are novel pre-rRNA binding proteins. While previous studies (7) suggested that these proteins bind directly each other, our data suggest that they also interact through an RNA intermediate. Further investigation of these candidates using techniques such as UV-crosslinking combined with immunoprecipitation and sequencing could be used to define the sites of pre-rRNA interaction.

Importantly, the RNAPI RNA interactome provides a new tool to understand how RNA-protein interactions control ribosome biogenesis. These data are of considerable interest since there are a number of disorders that are associated with dysregulated ribosome biogenesis. These include leukaemias (e.g. T-ALL, CLL), solids tumours (e.g. colon, gastric) and a group of rare syndromes termed ribosomopathies, many of which are associated with an increased cancer risk ((33,34). Ribosome biogenesis has been identified as a pathway that can be targeted in tumorigenesis and small molecule inhibitors are currently under development (e.g. CX5461 (27,28)). Thus, it is reasonable to predict that the RBPs required for ribosome biogenesis could provide new therapeutic avenues to identify novel chemicals that target this process.

## DATA AVAILABILITY

Code used in this study and supplementary data can be accessed via Bitbucket using this link https://bitbucket.org/emm13/2018_pineiro_nucleolarrbp/src.

## Supplementary Material

Supplementary Data
