# Supplementary material for: Identification of the RNA polymerase I-RNA interactome
Source: Nucleic Acids Res. 2018 Aug 30;46(20):11002–13. doi: 10.1093/nar/gky779 (PMC6237751; doi:10.1093/nar/gky779)
Supplement: Supplementary Data [file gky779_supplemental_files.zip › Revised supplementary Figures.pptx]

## Slide 1
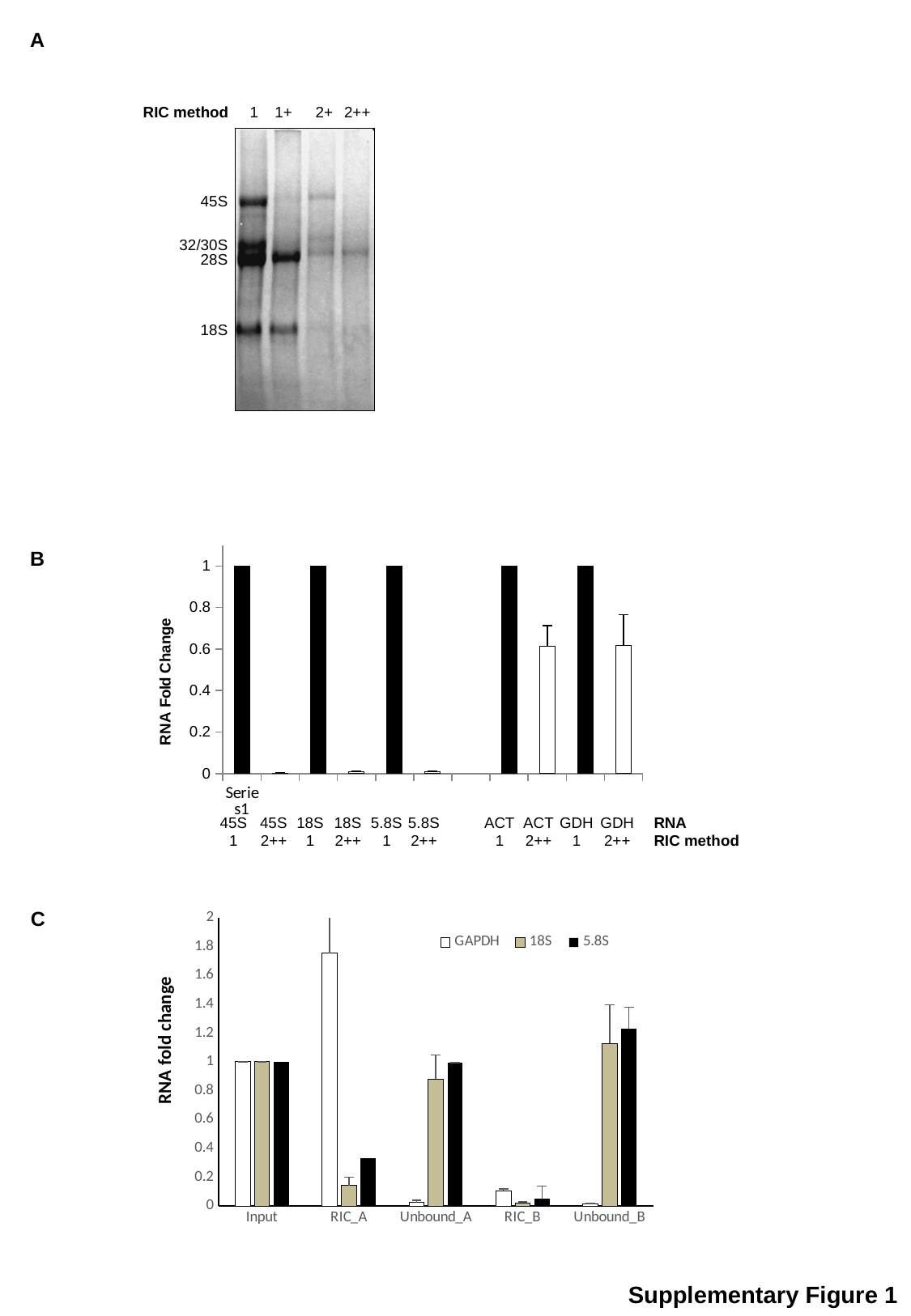

A
RIC method
1
1+
2+
2++
45S
32/30S
28S
18S
B
### Chart
| Category | |
|---|---|
| | 1.0 |
| | 0.0019352812775399618 |
| | 1.0 |
| | 0.00839448498458927 |
| | 1.0 |
| | 0.008477363317994669 |
| | None |
| | 1.0 |
| | 0.6145189277294695 |
| | 1.0 |
| | 0.6158254894853 |45S
1
45S
2++
18S
1
18S
2++
5.8S
1
5.8S
2++
ACT
1
ACT
2++
GDH
1
GDH
2++
RNA
RIC method
C
### Chart
| Category | GAPDH | 18S | 5.8S |
|---|---|---|---|
| Input | 1.0 | 1.0 | 1.0 |
| RIC_A | 1.753927291389628 | 0.14068152677791992 | 0.3336430071598489 |
| Unbound_A | 0.023275243544504293 | 0.8802240654629788 | 0.9945777618482333 |
| RIC_B | 0.10605993111717744 | 0.01997904144448813 | 0.05241179702550167 |
| Unbound_B | 0.01216551564988376 | 1.126203943163204 | 1.2283737854419607 |RNA fold change
Supplementary Figure 1

## Slide 2
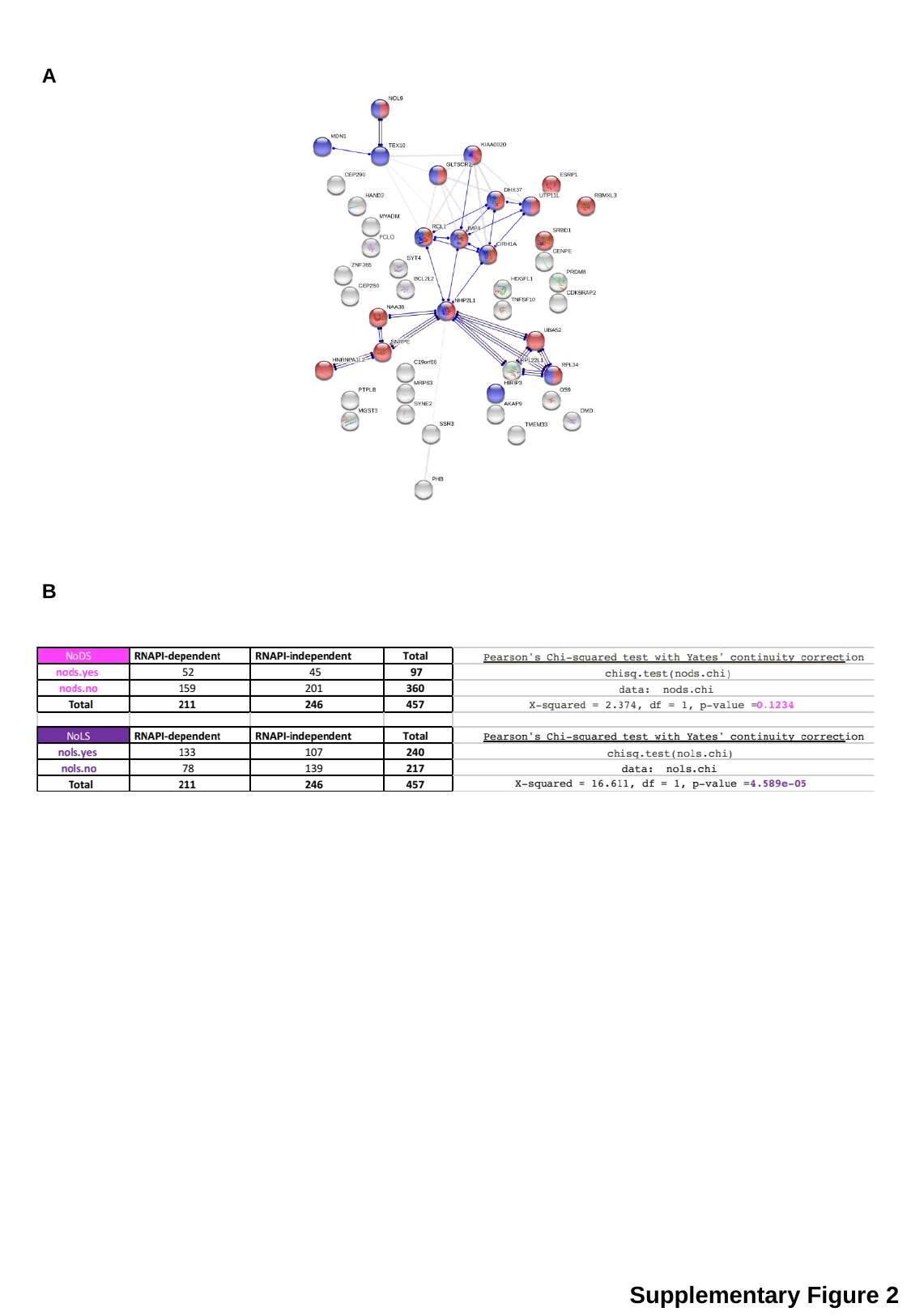

A
B
Supplementary Figure 2

## Slide 3
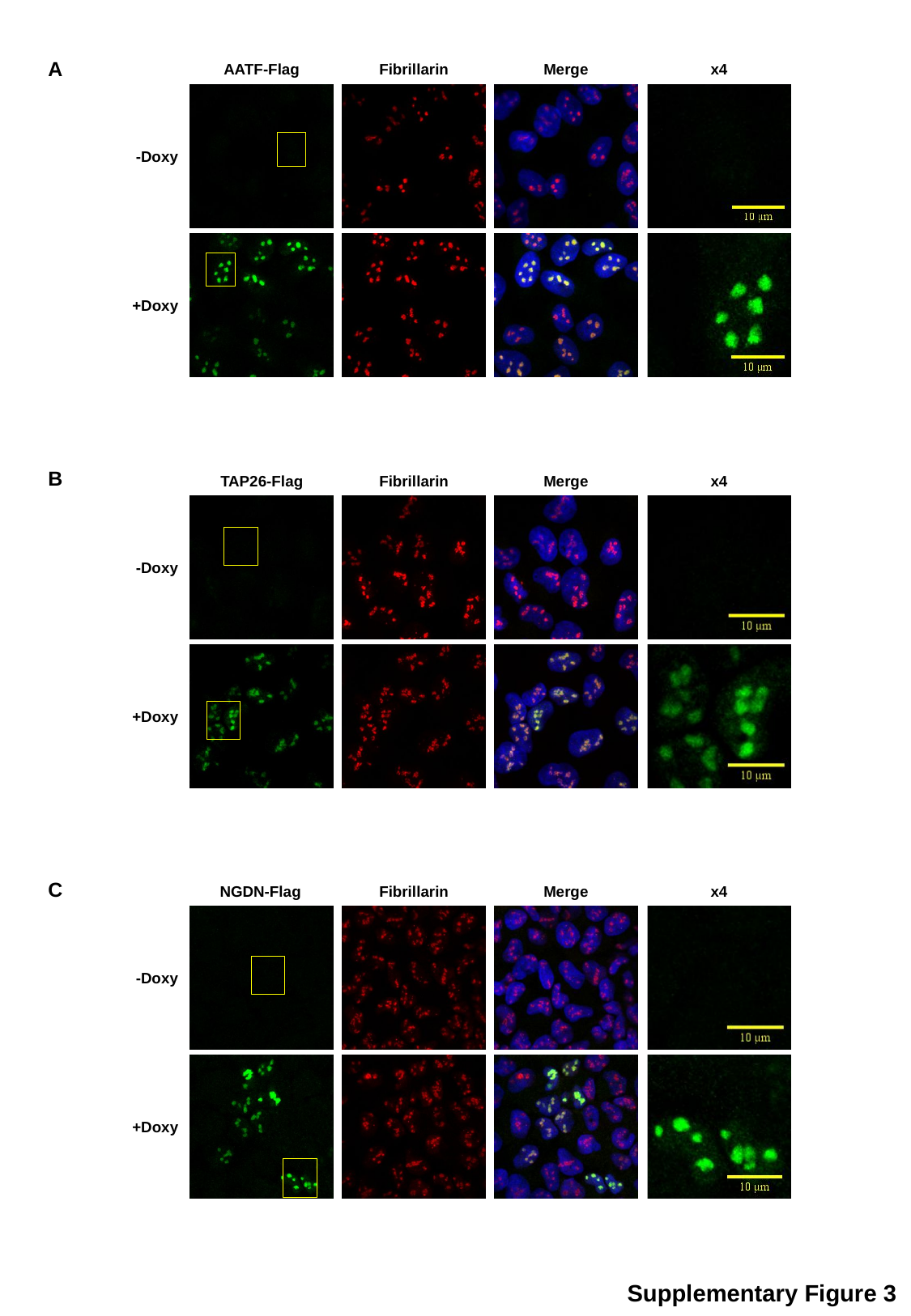

A
AATF-Flag
Fibrillarin
Merge
x4
-Doxy
+Doxy
TAP26-Flag
Fibrillarin
Merge
x4
-Doxy
+Doxy
NGDN-Flag
Fibrillarin
Merge
x4
-Doxy
+Doxy
B
C
Supplementary Figure 3

## Slide 4
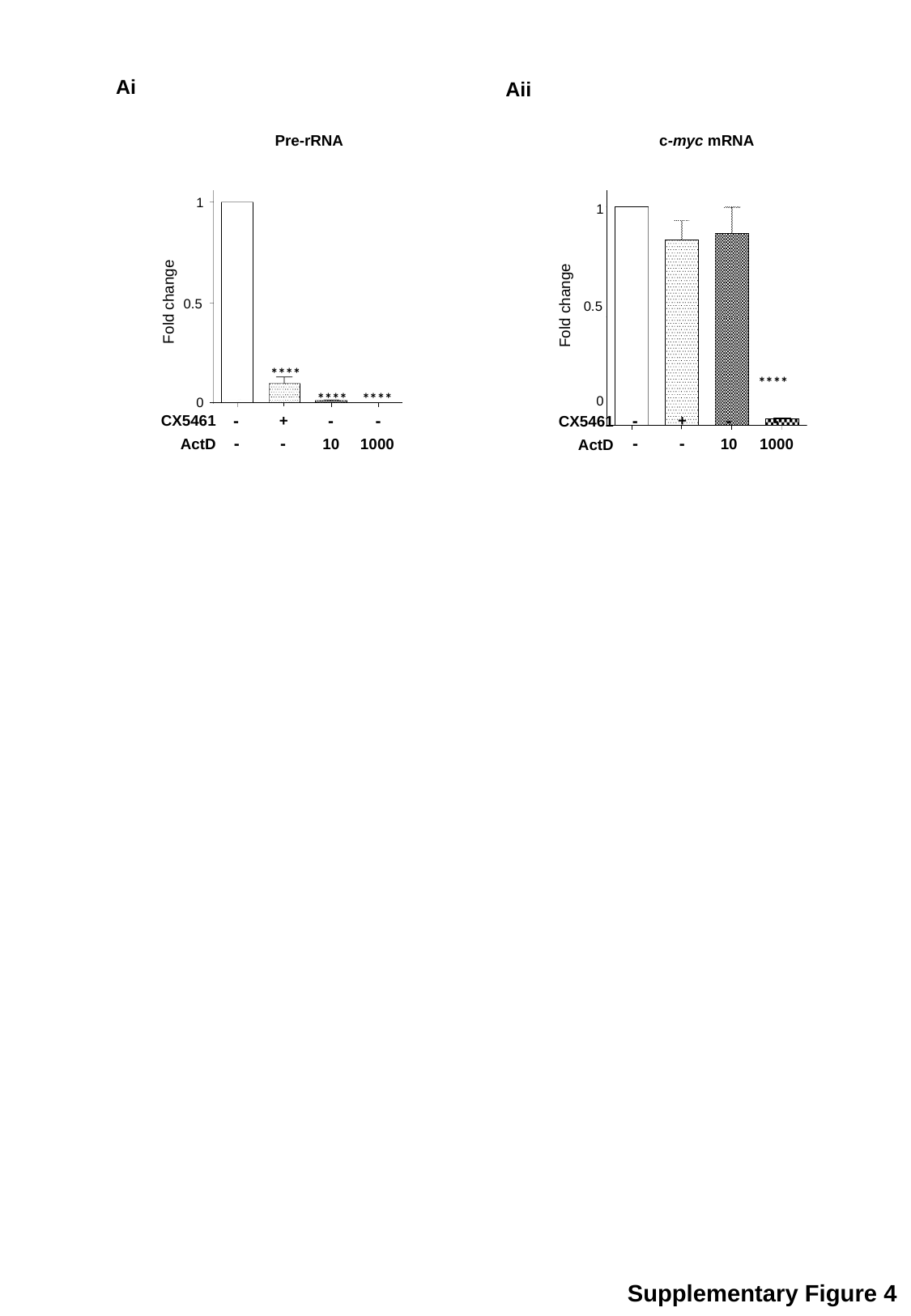

Ai
Aii
Pre-rRNA
1
Fold change
0.5
****
****
****
0
-
-
-
CX5461
+
-
-
ActD
10
1000
c-myc mRNA
1
Fold change
0.5
****
0
-
-
-
+
CX5461
-
-
10
1000
ActD
Supplementary Figure 4
