# Supplementary material for: Identification of the RNA polymerase I-RNA interactome
Source: Nucleic Acids Res. 2018 Aug 30;46(20):11002–13. doi: 10.1093/nar/gky779 (PMC6237751; doi:10.1093/nar/gky779)
Supplement: Supplementary Data [file gky779_supplemental_files.zip › Supplementary Figure Legends revised.docx]

**SUPPLEMENTARY MATERIAL**

**Supplementary Figure Legends**

***Supplementary Figure 1: Ribosomal RNA species are isolated during RIC as a result of RNA-RNA interactions between the mRNA and the abundant rRNA***

**(A)** RNA interactome capture was performed under the following conditions: one oligo(dT) isolation (RIC method 1), one oligo(dT) isolation with denaturation of the lysate (RIC method 1+), two sequential oligo(dT) affinity isolations with denaturation of the RNA from the first isolation (RIC method 2+), and two sequential oligo(dT) affinity isolations with denaturation of the lysate and the RNA from the first isolation (RIC method 2++). Denaturation was performed as described (1) RNA was resolved in a denaturing agarose gel, transferred to a membrane and stained with methylene blue. Ribosomal RNA species are abundant in the RNA from one oligo(dT) isolation (RIC method 1). Introducing two sequential oligo(dT) isolations with denaturation of the lysate and RNA from the first isolation (RIC method 2++) substantially depletes ribosomal RNA. **(B)** qRT-PCR analysis of the one-step (RIC method 1) and denaturing two-step (RIC method 2++) RIC RNA prepared as described (A). Sequences were amplified from the 5’ETS of the 45S pre-rRNA (45S), the 18S rRNA (18S), the 5.8S rRNA (5.8S), the actin mRNA (ACT) and the GAPDH mRNA (GDH). Ribosomal RNA species are vastly depleted using the denaturing two-step RIC method, whereas the majority of mRNA is captured using this method. (C) qRT-PCR analysis of two sequential oligo(dT) affinity isolations in native conditions. Purification of rRNA hybridised to polyadenylated mRNA with first round of RIC (RIC_A). The unbound fraction of RIC_A (Unbound_A) is subjected to second native RIC_B showing vast depletion of mRNA and subsequently loss of rRNA binding upon loss of mRNA.

***Supplementary Figure 2: Confirmation of RBPs and nucleolar proteins in our dataset***

**(A)** Functional enrichment and co-expression analysis using the STRING database (2) of the 48 candidate RBPs that were not identified in the HeLa, Huh or 293 RNA interactomes (Red = GO: RNA-binding; Blue = GO: Nucleolus).

**(B – top panel)** Nucleolar Detention Signal (NoDS) were searched for using the following consensus sequence {[RR(I/L)X_3_r]_(n,n>=1)_+[L(Phi/N)(V/L)]_(n,n>1)_}, where red represents the STAD motif and blue represents the STHD motif with “Phi” representing any hydrophobic residues including V, I, L, F, W, Y and M. For a protein to be NoDS positive, it should to contain at least 1 STAD and 2 or more STHD signals. Our set of proteins was annotated with predicted NoDS and a Chi-square test was performed to determine if there was a significant difference in the number of RNAPI-dependent proteins containing NoDS relative to the RNAPI-independent ones. **(B – bottom panel)** The NoLS database maintained by the Barton Group (http://www.compbio.dundee.ac.uk/www-nod/) was used to download a pre-annotated, predicted set of NoLSs from 9531 human proteins. Our set of proteins was annotated with predicted NoLS and a Chi-square test was performed to determine if there was a significant difference in the number of RNAPI-dependent proteins containing NoLS relative to the RNAPI-independent ones.

***Supplementary Figure 3: Inducible expression of FLAG-tagged proteins in U2OS cell lines***

Stable cell lines expressing FLAG-tagged AATF (A), TAP26 (B) and NGDN (C) proteins under the control of a tetracycline-inducible promoter were generated using the Flp-in T-REx system. Following induction with doxycycline, the expression and subcellular distribution of these proteins was monitored using immunofluorescence. FLAG antibody was used to identify the tagged proteins, Hoechst staining was used to as a nuclear marker and fibrillarin as a nucleolar marker. The data show that all three proteins are expressed after addition of doxycycline and are predominately localised in the nucleolus. The size bar represents 10 microns.

***Supplementary Figure 4: Treatment of cells with 10 nM ActD or CX5461 inhibits rRNA synthesis but not mRNA synthesis***

**(Ai)** RNA was isolated from U2OS cells treated with ActD at 10 nM or 1 µM, or with CX5461 at 2.5 µM. RT-qPCR was performed on RNA samples using primers to amplify sequence from the pre-rRNA 5’ETS to measure the inhibition of rRNA transcription. Actinomycin D and CX5461 treatment substantially reduces the cellular abundance of pre-rRNA. **(Aii)** RNA was prepared as in (Ai) and RT-qPCR was performed on the RNA using primers to amplify c-*myc* mRNA sequence – the c-*myc* mRNA has a half-life of approximately 20 minutes and is therefore a good readout for RNAPII activity. CX5461 and 10 nM actinomycin D treatment has no effect on c-*myc* mRNA abundance, but 1 µM actinomycin D significantly reduces c-*myc* RNA levels. The data represent the mean ± SD of three independent experiments, **** (p 0.0005), ** (p˂0.005) and * (p˂0.05).

***Supplementary Tables***

A full complement of data generated in this study and comparative analyses. The tables are numbered S1-S7 and their contents are described in the first sheet titled “Descriptions”.

***Supplementary References***

1. Pinol-Roma, S., Swanson, M.S., Matunis, M.J. and Dreyfuss, G. (1990) Purification and characterization of proteins of heterogeneous nuclear ribonucleoprotein complexes by affinity chromatography. *Methods in enzymology*, **181**, 326-331.

2. Szklarczyk, D., Franceschini, A., Wyder, S., Forslund, K., Heller, D., Huerta-Cepas, J., Simonovic, M., Roth, A., Santos, A., Tsafou, K.P. *et al.* (2015) STRING v10: protein-protein interaction networks, integrated over the tree of life. *Nucleic acids research*, **43**, D447-452.
